# [1,3-Bis(2-ethoxy­phen­yl)triazenido]bromidomercury(II)

**DOI:** 10.1107/S1600536809038732

**Published:** 2009-09-30

**Authors:** Mohammad Kazem Rofouei, Armin Beiza, Jafar Attar Gharamaleki

**Affiliations:** a Faculty of Chemistry, Tarbiat Moallem University, Tehran, Iran; b Young Researchers Club, Islamic Azad University, North Tehran Branch, Tehran, Iran

## Abstract

To the central atom of the title compound, [HgBr(C_16_H_18_N_3_O_2_)], is attached one bromide ion and a 1,3-bis­(2-ethoxy­phen­yl)triazenide ligand through one O and two N atoms, forming a distorted square-planar geometry around the Hg^II^ atom. The mononuclear complexes are linked into centrosymmetric dimers by non-classical inter­molecular C—H⋯N hydrogen bonds and by weak Hg–η^3^-arene π-inter­actions [mean distance = 3.434 (3) Å]. The resulting dimeric units are assembled into zigzag chains by translation along the crystallographic *c* axis through secondary C—H⋯π edge-to-face benzene ring inter­actions.

## Related literature

For aryl triazenes, their structural properties and metal complexes see: Vrieze & Van Koten (1987 ▶); Hörner *et al.* (2002 ▶, 2004 ▶, 2006 ▶). For the different coordination modes of the triazenide ligand, see: Moore & Robinson (1986 ▶). For the synthesis and mol­ecular structure of similar structures with cyano, meth­oxy and eth­oxy groups, see: Melardi *et al.* (2008 ▶); Rofouei *et al.* (2006 ▶); Rofouei, Melardi, Salemi *et al.* (2009 ▶). For the synthesis and crystal structures of Hg^II^ complexes with [1,3-bis­(2-methoxy­phen­yl)]triazene by using HgCl_2_, HgBr_2_, Hg(CH_3_COO)_2_ and Hg(SCN)_2_ salts as starting materials, see: Melardi *et al.* (2007 ▶); Hematyar & Rofouei (2008 ▶); Rofouei, Hematyar *et al.* (2009 ▶). For the synthesis and crystal structures of cadmium(II) and silver(I) complexes with 1,3-bis­(2-methoxy­phen­yl)triazene, see: Rofouei, Melardi, Khalili Ghaydari *et al.* (2009 ▶) and Payehghadr *et al.* (2007 ▶), respectively. For the synthesis and characterization of an isomorphous Hg^II^ structure with [1,3-bis­(2-ethoxy­phen­yl)]triazene by using HgCl_2_ instead of HgBr_2_, see: Melardi *et al.* (2009 ▶).
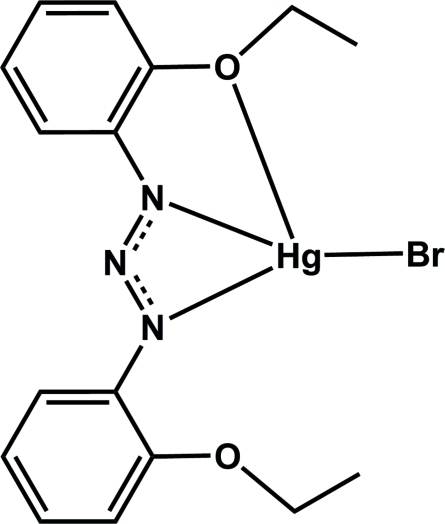


## Experimental

### 

#### Crystal data

[HgBr(C_16_H_18_N_3_O_2_)]
*M* *_r_* = 564.83
Monoclinic, 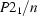

*a* = 10.2359 (7) Å
*b* = 7.4659 (5) Å
*c* = 22.4123 (14) Å
β = 98.860 (6)°
*V* = 1692.32 (19) Å^3^
*Z* = 4
Mo *K*α radiation
μ = 11.47 mm^−1^
*T* = 100 K
0.28 × 0.12 × 0.03 mm

#### Data collection

Bruker APEXII CCD diffractometer
Absorption correction: multi-scan (*SADABS*; Sheldrick, 1998 ▶) *T*_min_ = 0.221, *T*_max_ = 0.709
20759 measured reflections
4943 independent reflections
4081 reflections with *I* > 2σ(*I*)
*R*_int_ = 0.061

#### Refinement

*R*[*F* ^2^ > 2σ(*F* ^2^)] = 0.029
*wR*(*F* ^2^) = 0.062
*S* = 1.00
4943 reflections
210 parameters
H-atom parameters constrained
Δρ_max_ = 1.33 e Å^−3^
Δρ_min_ = −1.43 e Å^−3^

### 

Data collection: *APEX2* (Bruker, 2005 ▶); cell refinement: *SAINT* (Bruker, 2005 ▶); data reduction: *SAINT*; program(s) used to solve structure: *SHELXTL* (Sheldrick, 2008 ▶); program(s) used to refine structure: *SHELXTL*; molecular graphics: *Mercury* (Macrae *et al.*, 2006 ▶), *PLATON* (Spek, 2009 ▶) and *SHELXTL* (Sheldrick, 2008 ▶); software used to prepare material for publication: *SHELXTL*.

## Supplementary Material

Crystal structure: contains datablocks I, global. DOI: 10.1107/S1600536809038732/om2271sup1.cif

Structure factors: contains datablocks I. DOI: 10.1107/S1600536809038732/om2271Isup2.hkl

Additional supplementary materials:  crystallographic information; 3D view; checkCIF report

## Figures and Tables

**Table 1:** Hydrogen-bond geometry (Å, °)

*D*—H⋯*A*	*D*—H	H⋯*A*	*D*⋯*A*	*D*—H⋯*A*
C13—H13*B*⋯N2^i^	0.99	2.60	3.496 (5)	151
C12—H12⋯*Cg*1^ii^	0.95	2.85	3.559 (4)	132
C13—H13*A*⋯*Cg*1^iii^	0.99	2.72	3.523 (4)	139

Symmetry codes: (i) 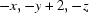; (ii) 
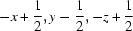
; (iii) 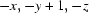.  *Cg*1 is the centroid of the C7–C12 aromatic ring.

**Fig. 1.**
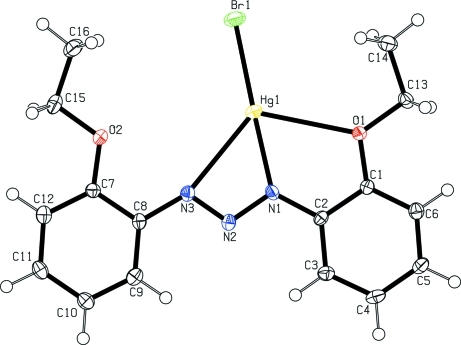
Molecular structure of the title compound, with ellipsoids drawn at the 50% probability level.

**Fig. 2.**
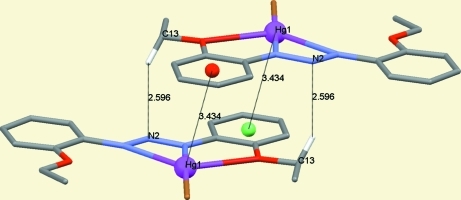
Non-classical C13–H13B···N2 hydrogen bond with H13B···N2 distance of 2.60 Å and weak Hg-η3-arene π-interactions (mean distance of 3.434 (3) Å) between dimers. The secondary Hg-η3-arene π-interactions involve three carbon atoms of the C1–C6 phenyl ring.

**Fig. 3.**
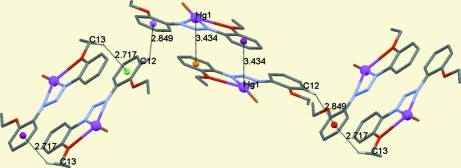
C–H···π stacking interactions between CH group of phenyl rings and aromatic rings with H···π distances of 2.72 and 2.85 Å for C13–H13A···Cg1 and C12–H12···Cg1 (Cg1 is the centroid for the C7—C12 aromatic ring).

**Fig. 4.**
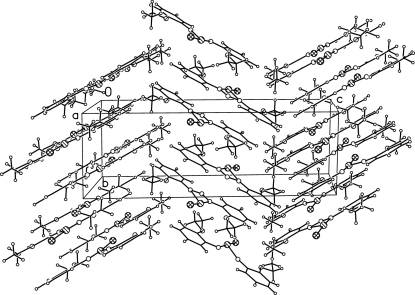
The packing diagram of the title compound.

## supplementary crystallographic information

### Comment

Transition metal complexes containing 1,3-diaryltriazenide ligands have been the object of numerous structural studies in the past few years because of their diverse range of coordination geometries. The uncoordinated –NN–N– compounds commonly adopt a *trans* configuration in the ground state. As ligands they exhibit versatile coordination geometries: with single or twofold nitrogen chains, neutral or anionic (triazenides) donor sets; they can be monodentate, (N1, N3)-chelating towards one metal atom or (N1, N3)-bridging over two metal atoms (Moore & Robinson, 1986), and they show a remarkable ability to support the stereochemical requisites of a wide variety of transition metal complexes (Hörner *et al.*, 2002, 2004). In these compounds, secondary bonds, or interactions such as hydrogen bonds and metal π-aryl interactions, can play an important role in their structures (Vrieze & Van Koten, 1987; Hörner *et al.*, 2006). We have previously reported the synthesis of the ligands, 1,3-bis(2-methoxyphenyl)]triazene (Rofouei *et al.*, 2006), [1,3-bis(2-ethoxyphenyl)]triazene (Rofouei, Melardi, Salemi *et al.*, 2009), and [1,3-bis(2-cyanophenyl)]triazene (Melardi *et al.*, 2008). Representative metal complexes include the Hg^II^ complex with [1,3-bis(2-methoxyphenyl)]triazene by using HgCl_2_ (Melardi *et al.*, 2007), HgBr_2_ (Hematyar & Rofouei 2008), Hg(CH_3_COO)_2_ and Hg(SCN)_2_ (Rofouei, Hematyar *et al.*, 2009) salts as starting materials. In addition, Ag(I) and Cd(II) complexes with this ligand are known (Payehghadr *et al.*, 2007; Rofouei, Melardi, Khalili Ghaydari *et al.*, 2009). More recently, a Hg^II^ complex with [1,3-bis(2-ethoxyphenyl)]triazene as ligand was reported in which HgCl_2_ was used as the starting salt (Melardi *et al.*, 2009). In this paper, a Hg^II^ complex using HgBr_2_ as a starting material is reported. It is isomorphous to the latter chloride complex.

The molecular structure of HgBr(C_16_H_18_N_3_O_2_) is presented in Fig. 1. The Hg^II^ atom is coordinated by one triazenide and one bromide ion. The 1,3-bis(2-ethoxyphenyl)triazenide is coordinated to the central atom through two N atoms [Hg1–N1 = 2.086 (3) Å and Hg1–N3 = 2.660 (3) Å] and one O atom [Hg1–O1 = 2.722 (3) Å] at a relatively long distance. The Hg1–Br1 distance of 2.4014 (4) Å is shorter than the corresponding distance in 1,3-bis(2-methoxyphenyl) with Hg1–Br1 = 2.5175 (11) Å (Hematyar & Rofouei, 2008). The Hg–Cl, Hg–O and Hg–N bonds distances in the isomorphous chloride, HgCl(C_16_H_18_N_3_O_2_), are 2.284 (8), 2.721 (2), 2.074 (2) and 2.674 (2) Å, respectively. The atoms of the ligand and the bromide ion generate a plane (maximum deviation from coplanarity of 0.037 Å).

In the title compound, the monomeric HgBr(C_16_H_18_N_3_O_2_) moieties are linked to pairs through non-classical C13–H13B···N2 hydrogen bond (C13···N2 = 3.496 (5) Å and <C13–H13B···N2 = 151 °, symmetry code (-*x*, 2 - *y*, -*z*). Also, weak Hg-η^3^-arene π-interactions (mean distance of 3.434 (3) Å) are present between these dimers. The secondary Hg-η^3^-arene π-interactions involving three carbon atoms of the C1–C6 phenyl ring. These metal-π interactions involve the Hg1 atom and C4 [3.461 (5) Å], C5 [3.254 (5) Å] and C6 [3.515 (5) Å] atoms with related symmetry code (-*x*, 2 - *y*, -*z*) (Fig. 2). The resulted dimeric units are assembled into zigzag chains by translation along the crystallographic *c* axis through secondary C–H···π stacking interactions. These edge-to-face interactions are present between CH group of phenyl rings and aromatic rings with H···π distances of 2.72 and 2.85 Å for C13–H13A···*Cg*1 [symmetry code: 1/2 - *x*, -1/2 + *y*, 1/2 - *z*] and C12–H12···*Cg*1 [symmetry code: -*x*, 1 - *y*, -*z*], respectively in which *Cg*1 is the centroid for C7—C12 aromatic ring (Fig. 3). The sum of the weak non-covalent interactions seems to play an important role in the crystal packing. The unit cell packing diagram of the title compound is shown in Fig. 4.

### Experimental

A methanol solution of 1,3-bis(2-ethoxyphenyl)triazene (0.2853 g, 1 mmol) was added to a methanol solution of mercury(II) bromide (0.3604 g, 1 mmol). After mixing for 1 h at room temperature, an orange solid was readily precipitated out. It was then filtered off, washed with methanol and dried in vacuum. The orange crude material was dissolved in 10 ml of THF, and placed in a freezer without covering. After two weeks beautiful orange and air-stable crystals of the title complex were obtained by slow evaporation of the solvent.

### Refinement

Positions of the H(C) were calculated from geometry with C—H = 0.95 - 0.99 Å. All hydrogen atoms were refined by use of a riding model with *U*_iso_(H) parameters equal to 1.5 *U*_eq_(C) for methyl groups and to 1.2 *U*_eq_(C) for other carbon atoms where *U*_eq_(C) are the equivalent isotropic thermal parameters of the atoms to which the corresponding H atoms are bonded.

### Crystal data

**Table:**

[HgBr(C_16_H_18_N_3_O_2_)]	*F*(000) = 1064
*M**_r_* = 564.83	*D*_x_ = 2.217 Mg m^−^^3^
Monoclinic, *P*2_1_/*n*	Mo *K*α radiation, λ = 0.71073 Å
Hall symbol: -P 2yn	Cell parameters from 4844 reflections
*a* = 10.2359 (7) Å	θ = 2.3–29.5°
*b* = 7.4659 (5) Å	µ = 11.47 mm^−^^1^
*c* = 22.4123 (14) Å	*T* = 100 K
β = 98.860 (6)°	Plate, orange
*V* = 1692.32 (19) Å^3^	0.28 × 0.12 × 0.03 mm
*Z* = 4	

### Data collection

**Table:**

Bruker APEXII CCD diffractometer	4943 independent reflections
Radiation source: fine-focus sealed tube	4081 reflections with *I* > 2σ(*I*)
graphite	*R*_int_ = 0.061
ω scans	θ_max_ = 30.0°, θ_min_ = 2.1°
Absorption correction: multi-scan (*SADABS*; Sheldrick, 1998)	*h* = −14→14
*T*_min_ = 0.221, *T*_max_ = 0.709	*k* = −10→10
20759 measured reflections	*l* = −31→31

### Refinement

**Table:**

Refinement on *F*^2^	Primary atom site location: structure-invariant direct methods
Least-squares matrix: full	Secondary atom site location: difference Fourier map
*R*[*F*^2^ > 2σ(*F*^2^)] = 0.029	Hydrogen site location: inferred from neighbouring sites
*wR*(*F*^2^) = 0.062	H-atom parameters constrained
*S* = 1.00	*w* = 1/[σ^2^(*F*_o_^2^) + (0.01*P*)^2^] where *P* = (*F*_o_^2^ + 2*F*_c_^2^)/3
4943 reflections	(Δ/σ)_max_ = 0.001
210 parameters	Δρ_max_ = 1.33 e Å^−^^3^
0 restraints	Δρ_min_ = −1.43 e Å^−^^3^

### Special details

**Table:**

Geometry. All e.s.d.'s (except the e.s.d. in the dihedral angle between two l.s. planes) are estimated using the full covariance matrix. The cell e.s.d.'s are taken into account individually in the estimation of e.s.d.'s in distances, angles and torsion angles; correlations between e.s.d.'s in cell parameters are only used when they are defined by crystal symmetry. An approximate (isotropic) treatment of cell e.s.d.'s is used for estimating e.s.d.'s involving l.s. planes.
Refinement. Refinement of *F*^2^ against ALL reflections. The weighted *R*-factor *wR* and goodness of fit *S* are based on *F*^2^, conventional *R*-factors *R* are based on *F*, with *F* set to zero for negative *F*^2^. The threshold expression of *F*^2^ > σ(*F*^2^) is used only for calculating *R*-factors(gt) *etc*. and is not relevant to the choice of reflections for refinement. *R*-factors based on *F*^2^ are statistically about twice as large as those based on *F*, and *R*- factors based on ALL data will be even larger. The maximum and minimum difference map peaks are within 1.21 Å of Hg1.

### Fractional atomic coordinates and isotropic or equivalent isotropic displacement parameters (Å^2^)

**Table:**

	*x*	*y*	*z*	*U*_iso_*/*U*_eq_	
Hg1	−0.152069 (14)	0.681795 (19)	0.053806 (7)	0.01739 (5)	
Br1	−0.36007 (4)	0.63210 (6)	0.08813 (2)	0.02803 (10)	
O1	−0.1764 (2)	0.8602 (3)	−0.05325 (12)	0.0160 (5)	
O2	0.0190 (3)	0.4368 (3)	0.20087 (13)	0.0175 (6)	
N1	0.0246 (3)	0.7115 (4)	0.01922 (15)	0.0148 (6)	
N2	0.1262 (3)	0.6390 (4)	0.05569 (15)	0.0140 (6)	
N3	0.0888 (3)	0.5769 (4)	0.10309 (15)	0.0147 (6)	
C1	−0.0555 (3)	0.8727 (5)	−0.07197 (17)	0.0127 (7)	
C2	0.0503 (4)	0.7918 (4)	−0.03385 (17)	0.0133 (7)	
C3	0.1769 (4)	0.7992 (5)	−0.05036 (18)	0.0170 (8)	
H3	0.2493	0.7441	−0.0254	0.020*	
C4	0.1972 (4)	0.8862 (5)	−0.1028 (2)	0.0202 (8)	
H4	0.2834	0.8906	−0.1136	0.024*	
C5	0.0928 (4)	0.9665 (5)	−0.13943 (18)	0.0182 (8)	
H5	0.1073	1.0270	−0.1751	0.022*	
C6	−0.0331 (4)	0.9589 (5)	−0.12414 (18)	0.0168 (8)	
H6	−0.1048	1.0134	−0.1497	0.020*	
C7	0.1494 (3)	0.4203 (5)	0.19634 (18)	0.0143 (7)	
C8	0.1896 (3)	0.4974 (5)	0.14479 (17)	0.0136 (7)	
C9	0.3235 (4)	0.4931 (5)	0.13872 (18)	0.0172 (8)	
H9	0.3514	0.5475	0.1045	0.021*	
C10	0.4158 (4)	0.4114 (5)	0.18149 (19)	0.0187 (8)	
H10	0.5066	0.4105	0.1770	0.022*	
C11	0.3744 (4)	0.3301 (5)	0.23141 (19)	0.0183 (8)	
H11	0.4371	0.2716	0.2606	0.022*	
C12	0.2414 (4)	0.3343 (5)	0.23868 (19)	0.0173 (8)	
H12	0.2139	0.2781	0.2727	0.021*	
C13	−0.2862 (4)	0.9515 (5)	−0.08961 (18)	0.0174 (8)	
H13A	−0.3002	0.9034	−0.1313	0.021*	
H13B	−0.2678	1.0814	−0.0914	0.021*	
C14	−0.4070 (4)	0.9196 (6)	−0.0602 (2)	0.0233 (9)	
H14A	−0.4809	0.9907	−0.0809	0.035*	
H14B	−0.3885	0.9552	−0.0177	0.035*	
H14C	−0.4303	0.7922	−0.0630	0.035*	
C15	−0.0256 (4)	0.3651 (5)	0.25327 (19)	0.0213 (8)	
H15A	0.0251	0.4179	0.2902	0.026*	
H15B	−0.0132	0.2336	0.2548	0.026*	
C16	−0.1692 (4)	0.4104 (6)	0.2491 (2)	0.0272 (10)	
H16A	−0.2048	0.3543	0.2828	0.041*	
H16B	−0.2173	0.3659	0.2108	0.041*	
H16C	−0.1795	0.5406	0.2511	0.041*	

### Atomic displacement parameters (Å^2^)

**Table:**

	*U*^11^	*U*^22^	*U*^33^	*U*^12^	*U*^13^	*U*^23^
Hg1	0.01495 (7)	0.01991 (8)	0.01826 (8)	−0.00083 (5)	0.00561 (5)	0.00169 (6)
Br1	0.01972 (19)	0.0300 (2)	0.0374 (3)	0.00079 (16)	0.01402 (18)	0.00757 (19)
O1	0.0130 (12)	0.0221 (13)	0.0132 (14)	0.0018 (10)	0.0027 (10)	0.0021 (11)
O2	0.0165 (13)	0.0211 (13)	0.0156 (14)	−0.0003 (10)	0.0042 (11)	0.0040 (11)
N1	0.0134 (14)	0.0167 (15)	0.0134 (16)	0.0014 (11)	−0.0006 (12)	0.0011 (12)
N2	0.0169 (15)	0.0129 (14)	0.0117 (16)	−0.0016 (11)	0.0006 (12)	−0.0013 (12)
N3	0.0156 (15)	0.0143 (14)	0.0138 (16)	−0.0030 (11)	0.0010 (12)	0.0007 (12)
C1	0.0149 (17)	0.0127 (15)	0.0103 (17)	−0.0005 (13)	0.0017 (14)	−0.0027 (14)
C2	0.0158 (17)	0.0123 (16)	0.0123 (18)	−0.0007 (12)	0.0043 (14)	−0.0009 (13)
C3	0.0142 (17)	0.0181 (18)	0.019 (2)	−0.0015 (13)	0.0032 (15)	0.0000 (15)
C4	0.0159 (18)	0.0242 (19)	0.022 (2)	−0.0039 (15)	0.0085 (16)	−0.0017 (17)
C5	0.0197 (19)	0.0212 (19)	0.0149 (19)	−0.0034 (15)	0.0061 (15)	0.0005 (16)
C6	0.0183 (18)	0.0148 (17)	0.017 (2)	−0.0002 (14)	0.0017 (15)	0.0008 (15)
C7	0.0141 (17)	0.0119 (16)	0.0168 (19)	0.0004 (13)	0.0028 (14)	0.0005 (14)
C8	0.0170 (17)	0.0123 (16)	0.0110 (18)	0.0001 (13)	0.0007 (14)	−0.0020 (14)
C9	0.0208 (19)	0.0146 (17)	0.017 (2)	−0.0034 (14)	0.0041 (15)	−0.0011 (15)
C10	0.0171 (18)	0.0187 (19)	0.019 (2)	−0.0005 (14)	−0.0001 (15)	−0.0015 (16)
C11	0.0183 (18)	0.0173 (17)	0.017 (2)	0.0004 (14)	−0.0035 (15)	−0.0022 (16)
C12	0.0215 (18)	0.0131 (17)	0.0167 (19)	−0.0001 (14)	0.0018 (15)	−0.0004 (15)
C13	0.0146 (17)	0.0189 (18)	0.017 (2)	−0.0005 (14)	−0.0018 (15)	0.0005 (16)
C14	0.0162 (19)	0.030 (2)	0.023 (2)	0.0012 (16)	0.0029 (16)	−0.0019 (18)
C15	0.029 (2)	0.0205 (19)	0.015 (2)	0.0008 (16)	0.0069 (17)	0.0025 (16)
C16	0.029 (2)	0.029 (2)	0.028 (3)	0.0044 (17)	0.0154 (19)	0.0074 (19)

### Geometric parameters (Å, °)

**Table:**

Hg1—N1	2.086 (3)	C7—C8	1.408 (5)
Hg1—Br1	2.4014 (4)	C8—C9	1.398 (5)
Hg1—N3	2.660 (3)	C9—C10	1.380 (5)
O1—C1	1.370 (4)	C9—H9	0.9500
O1—C13	1.452 (4)	C10—C11	1.395 (6)
O2—C7	1.359 (4)	C10—H10	0.9500
O2—C15	1.428 (5)	C11—C12	1.396 (5)
N1—N2	1.334 (4)	C11—H11	0.9500
N1—C2	1.393 (5)	C12—H12	0.9500
N2—N3	1.271 (4)	C13—C14	1.507 (5)
N3—C8	1.412 (5)	C13—H13A	0.9900
C1—C6	1.384 (5)	C13—H13B	0.9900
C1—C2	1.408 (5)	C14—H14A	0.9800
C2—C3	1.403 (5)	C14—H14B	0.9800
C3—C4	1.386 (6)	C14—H14C	0.9800
C3—H3	0.9500	C15—C16	1.497 (6)
C4—C5	1.381 (6)	C15—H15A	0.9900
C4—H4	0.9500	C15—H15B	0.9900
C5—C6	1.385 (5)	C16—H16A	0.9800
C5—H5	0.9500	C16—H16B	0.9800
C6—H6	0.9500	C16—H16C	0.9800
C7—C12	1.387 (5)		
			
N1—Hg1—Br1	175.94 (9)	C10—C9—C8	121.3 (4)
N1—Hg1—N3	52.01 (11)	C10—C9—H9	119.4
Br1—Hg1—N3	129.22 (7)	C8—C9—H9	119.4
C1—O1—C13	117.0 (3)	C9—C10—C11	119.3 (4)
C7—O2—C15	118.0 (3)	C9—C10—H10	120.4
N2—N1—C2	117.8 (3)	C11—C10—H10	120.4
N2—N1—Hg1	111.6 (2)	C10—C11—C12	120.4 (4)
C2—N1—Hg1	130.7 (2)	C10—C11—H11	119.8
N3—N2—N1	110.7 (3)	C12—C11—H11	119.8
N2—N3—C8	115.1 (3)	C7—C12—C11	120.1 (4)
N2—N3—Hg1	85.7 (2)	C7—C12—H12	119.9
C8—N3—Hg1	159.2 (3)	C11—C12—H12	119.9
O1—C1—C6	124.4 (3)	O1—C13—C14	107.3 (3)
O1—C1—C2	115.6 (3)	O1—C13—H13A	110.3
C6—C1—C2	120.0 (3)	C14—C13—H13A	110.3
N1—C2—C3	123.1 (3)	O1—C13—H13B	110.3
N1—C2—C1	118.3 (3)	C14—C13—H13B	110.3
C3—C2—C1	118.6 (3)	H13A—C13—H13B	108.5
C4—C3—C2	120.5 (4)	C13—C14—H14A	109.5
C4—C3—H3	119.7	C13—C14—H14B	109.5
C2—C3—H3	119.7	H14A—C14—H14B	109.5
C5—C4—C3	120.3 (4)	C13—C14—H14C	109.5
C5—C4—H4	119.9	H14A—C14—H14C	109.5
C3—C4—H4	119.9	H14B—C14—H14C	109.5
C4—C5—C6	120.0 (4)	O2—C15—C16	107.5 (3)
C4—C5—H5	120.0	O2—C15—H15A	110.2
C6—C5—H5	120.0	C16—C15—H15A	110.2
C1—C6—C5	120.6 (4)	O2—C15—H15B	110.2
C1—C6—H6	119.7	C16—C15—H15B	110.2
C5—C6—H6	119.7	H15A—C15—H15B	108.5
O2—C7—C12	124.2 (4)	C15—C16—H16A	109.5
O2—C7—C8	116.0 (3)	C15—C16—H16B	109.5
C12—C7—C8	119.8 (3)	H16A—C16—H16B	109.5
C9—C8—C7	119.0 (3)	C15—C16—H16C	109.5
C9—C8—N3	124.9 (3)	H16A—C16—H16C	109.5
C7—C8—N3	116.0 (3)	H16B—C16—H16C	109.5
			
N3—Hg1—N1—N2	1.4 (2)	C3—C4—C5—C6	−0.6 (6)
N3—Hg1—N1—C2	−178.7 (4)	O1—C1—C6—C5	179.5 (3)
C2—N1—N2—N3	177.6 (3)	C2—C1—C6—C5	0.1 (5)
Hg1—N1—N2—N3	−2.5 (3)	C4—C5—C6—C1	0.6 (6)
N1—N2—N3—C8	−179.9 (3)	C15—O2—C7—C12	1.7 (5)
N1—N2—N3—Hg1	1.8 (3)	C15—O2—C7—C8	−178.3 (3)
N1—Hg1—N3—N2	−1.37 (19)	O2—C7—C8—C9	176.9 (3)
Br1—Hg1—N3—N2	173.68 (16)	C12—C7—C8—C9	−3.1 (5)
N1—Hg1—N3—C8	−177.1 (7)	O2—C7—C8—N3	−2.1 (5)
Br1—Hg1—N3—C8	−2.1 (7)	C12—C7—C8—N3	177.9 (3)
C13—O1—C1—C6	−3.0 (5)	N2—N3—C8—C9	3.9 (5)
C13—O1—C1—C2	176.4 (3)	Hg1—N3—C8—C9	179.2 (5)
N2—N1—C2—C3	0.1 (5)	N2—N3—C8—C7	−177.2 (3)
Hg1—N1—C2—C3	−179.7 (3)	Hg1—N3—C8—C7	−1.9 (8)
N2—N1—C2—C1	−178.4 (3)	C7—C8—C9—C10	1.5 (5)
Hg1—N1—C2—C1	1.7 (5)	N3—C8—C9—C10	−179.6 (3)
O1—C1—C2—N1	−1.5 (5)	C8—C9—C10—C11	0.6 (6)
C6—C1—C2—N1	177.9 (3)	C9—C10—C11—C12	−1.2 (6)
O1—C1—C2—C3	179.8 (3)	O2—C7—C12—C11	−177.5 (3)
C6—C1—C2—C3	−0.7 (5)	C8—C7—C12—C11	2.5 (5)
N1—C2—C3—C4	−177.8 (3)	C10—C11—C12—C7	−0.3 (6)
C1—C2—C3—C4	0.7 (5)	C1—O1—C13—C14	−179.7 (3)
C2—C3—C4—C5	−0.1 (6)	C7—O2—C15—C16	176.5 (3)

### Hydrogen-bond geometry (Å, °)

**Table:**

*D*—H···*A*	*D*—H	H···*A*	*D*···*A*	*D*—H···*A*
C13—H13B···N2^i^	0.99	2.60	3.496 (5)	151
C12—H12···Cg1^ii^	0.95	2.85	3.559 (4)	132
C13—H13A···Cg1^iii^	0.99	2.72	3.523 (4)	139

Symmetry codes: (i) −*x*, −*y*+2, −*z*; (ii) −*x*+1/2, *y*−1/2, −*z*+1/2; (iii) −*x*, −*y*+1, −*z*.
